# The complete chloroplast genome sequences of *Cornus elliptica* (Cornaceae)

**DOI:** 10.1080/23802359.2021.1888333

**Published:** 2021-03-16

**Authors:** Hui Lu, Jun Tang, Diqiang Li

**Affiliations:** Environment and Protection, The Chinese Academy of Forestry, The Research Institute of Forest Ecology, Beijing, China

**Keywords:** Cornaceae, Cornus, complete chloroplast genome, Dendrobenthamia angustata, Cornus elliptica

## Abstract

*Cornus elliptica* is a species of the *Cornus*, an evergreen tree endemic to China. Here, we report a complete chloroplast genome sequence of *C. elliptica*. The chloroplast genome was found to be 157,400bp in length, and G-C contents were 38.1%. The sequence contained 115 unique genes, including 31 tRNA, 4 rRNA, and 80 protein-coding genes. Phylogenetic framework of *Cornus* is consistent with previous studies.

*Cornus* L. is a genus of many economically valuable plants that are used for food, industry, and garden decoration and are widely distributed in temperate and subtropical regions of the Northern Hemisphere. Although the monophyly of *Cornus sensu lato* was strongly supported by morphological and molecular data, the taxonomic composition, and relationships of subgroups within the genus have been controversial for a long time (Xiang et al. 2006). In other words, there are two views on the division of *Cornus*, namely the broad view and the subdivision view. In the first treatment, genus has been either divided into different numbers (4–10) of sub-genera or sections by researchers (Eyde 1987; Xiang et al. 1998). However, some scholars treated it as several (up to 6) distinct genera based on their significant differences in morphological characteristics. *C. elliptica* (Pojarkova) Q. Y. Xiang & Boufford is a species of the *Cornus*, an evergreen tree endemic to China. The name *Dendrobenthamia angustata* (Chun) W.P. Fang recorded in FRPS of this species is invalid since the validly published synonym *Cynoxylon elliptica* Pojarkova (Yu 2006). Here, we report the complete chloroplast genome sequence of *Cornus elliptica* for the first time, which will provide genomic information to further reveal the phylogenetic relationship of Cornus.

Total genomic DNA was extracted from silica-dried leaves collected from Wufeng, Hubei (N30°05′43″, E110°37′10″) using a modified CTAB method (Doyle and Doyle 1987). A voucher specimen (collection number: J. Tang, CAF202000847) was collected and deposited in the Dendrological Herbarium, Chinese Academy of Forestry (CAF). DNA libraries preparation and pair-end 150-bp read length sequencing were performed on the Illumina HiSeq 4000 platform at Biomarker Technologies Corporation (http://en.biomarker.com.cn, China) and about 6 GB of clean data were trimmed. We used Map function of Geneious R11 (Kearse et al. 2012) to extract chloroplast reads using published chloroplast genome from *Cornus officinalis* (MN380657) as reference. Filtered chloroplast reads were used for *de novo* assembly with Geneious R11. Gaps were bridged using Fine Tuning function of Geneious R11. The three chloroplast sequences were then annotated using Plann (Huang and Cronk 2015), and annotations were verified by Geneious R11.

The plastome of *Cornus elliptica* was 157,400 bp in length containing a Large Single-Copy (LSC) region of 86,714 bp, a Small Single-Copy (SSC) region of 18,432 bp, and two Inverted Repeats (IR) of 26,127 bp. The genome contained 115 unique genes, including 80 protein-coding genes, 31 tRNA genes, and 4 rRNA genes. The sequence GC content was 38.1%.

Bayesian inference (BI) tree based on complete chloroplast genome sequences of 8 other *Cornus* species and *Alangium kurzii* as the outgroup from NCBI was reconstructed in MrBayes 3.2.3 (Ronquist and Huelsenbeck 2003). All the sequences were aligned by MAFFT v6.833 (Katoh 2005), and the appropriate nucleotide substitution model was chosen by using jModeltest (Posada 2008). The posterior probability (PP) of all branch nodes in this phylogenetic tree (Figure 1) is 1.00. Phylogenetic framework of *Cornus* is consistent with previous studies.

**Figure 1.**
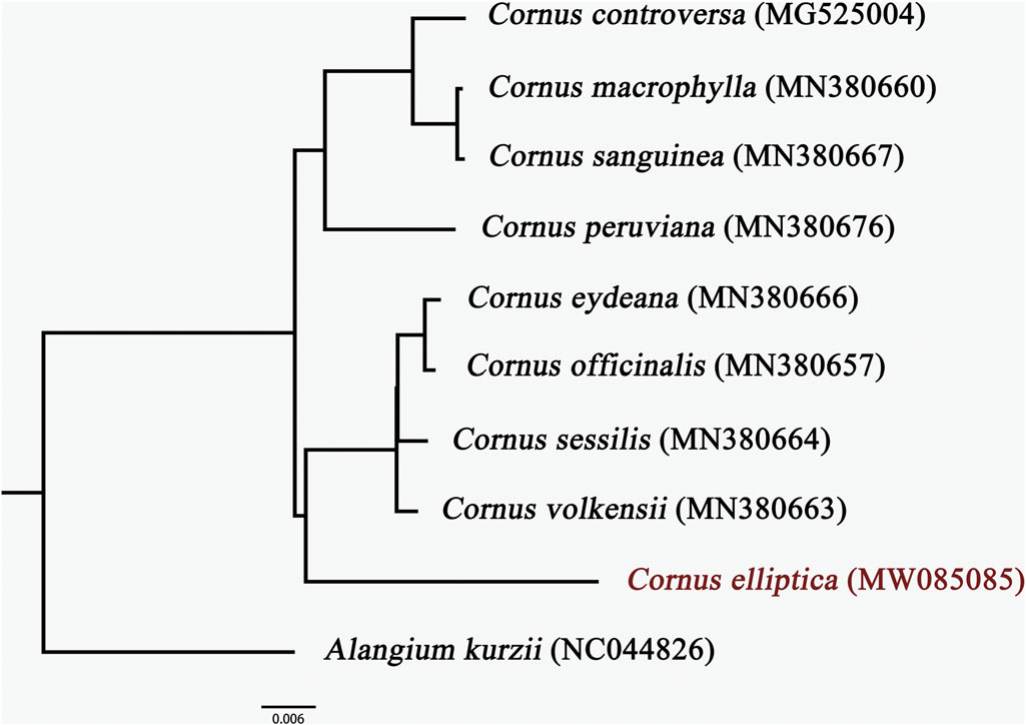
Bayesian phylogram of 9 Cornaceae species was reconstructed based on complete chloroplast genome sequences using *Alangium kurzii* as an outgroup. The posterior probability (PP) of all branch nodes in this phylogenetic tree is 1.00.

## Data Availability

The genome sequence data that support the findings of this study are openly available in GenBank of NCBI at (https://www.ncbi.nlm.nih.gov/) under the accession no. MW085085. The associated BioProject and SRA numbers are PRJNA666496, SRR12744696, respectively.
